# Species Choice for Comparative Genomics: Being Greedy Works

**DOI:** 10.1371/journal.pgen.0010071

**Published:** 2005-12-02

**Authors:** Fabio Pardi, Nick Goldman

**Affiliations:** EMBL-European Bioinformatics Institute, Wellcome Trust Genome Campus, Hinxton, United Kingdom; Princeton University, United States of America

## Abstract

Several projects investigating genetic function and evolution through sequencing and comparison of multiple genomes are now underway. These projects consume many resources, and appropriate planning should be devoted to choosing which species to sequence, potentially involving cooperation among different sequencing centres. A widely discussed criterion for species choice is the maximisation of evolutionary divergence. Our mathematical formalization of this problem surprisingly shows that the best long-term cooperative strategy coincides with the seemingly short-term “greedy” strategy of always choosing the next best single species. Other criteria influencing species choice, such as medical relevance or sequencing costs, can also be accommodated in our approach, suggesting our results' broad relevance in scientific policy decisions.

## Synopsis

What would happen if sequencing centres around the world were to choose genomes without consulting each other and without devising long-term strategies? When several parties are involved in decisions with interacting consequences, experience teaches that cooperation and planning are usually necessary to guarantee the best result. Similarly, in computer science, “greedy” algorithms—which construct solutions by iteratively taking the best immediate choice—are rarely the best option to solve a problem. The authors show, however, that in the context of choosing species for comparative genomics, cooperation and planning can be kept to a minimum without affecting the quality of the global result: a greedy algorithm applied to the problem of maximising the evolutionary divergence among species chosen from a known phylogeny is proven to guarantee optimal solutions.

## Introduction

Comparing biological sequences has enormous potential for increasing our knowledge about their function, structure, and evolution, an idea that has been applied virtually everywhere in computational biology. Comparative studies are now performed on a genomic scale, requiring the sequencing of entire genomes [1,2] or significant parts of them [3]. Choosing the right species for sequencing is therefore crucial. This involves two distinct decisions: first a range of species over which comparisons will be made is identified, and then a number of them are selected for actual sequencing. The first decision specifies what is known as the phylogenetic scope [4] or lineal scope [5] and is made largely on the basis of the biology the species are required to share. Different research communities are focusing on different scopes—for example, yeasts [6], nematodes [7], fruit flies [8], mammals [9], and primates [10]—corresponding to the investigation of functional elements of different biological importance.

In this article, we deal with the second decision: selecting the genomes to sequence from the chosen scope. Although this decision is determined by a variety of factors [11], chief among them is the objective of maximising the evolutionary divergence among the chosen species: the more diverse the genomes being compared, the more we can observe the different paths taken by evolution and learn about the features common to all species in the phylogenetic scope. Maximising evolutionary divergence has, for example, been advocated as a way to attain maximum sensitivity in the detection of conserved genomic regions [3,12]—regions that accumulate substitutions at a rate significantly lower than the genome-wide average. These regions are likely to be functional, as the simplest explanation for this phenomenon is the action of purifying selection (for example, see [1,3,10]), and the characterisation of non-coding conserved regions is of particular interest because their function remains unclear [9,13]. Although a maximally divergent set of species does not necessarily guarantee maximum statistical power for detecting evolutionary conservation [5], it is probably advantageous for all practical phylogenetic scopes: counterexamples are likely to arise only for (evolutionarily) very wide phylogenetic scopes, which are unrealistic in practice due to the resulting difficulty of alignment [12] and the pooling of species with different biologies.

Formalizing the problem of selecting species to maximise divergence is straightforward. Consider a phylogenetic tree connecting all the species in the chosen scope, with branch lengths representing the amount of molecular evolution between nodes in the tree. The divergence of a set of species is defined as the total branch length of the subtree connecting them (Figure 1A). The problem then becomes: given that we have already sequenced some species, and now have resources to sequence *k* additional species, which should we choose in order to maximise the divergence of the resulting set?

**Figure 1:**
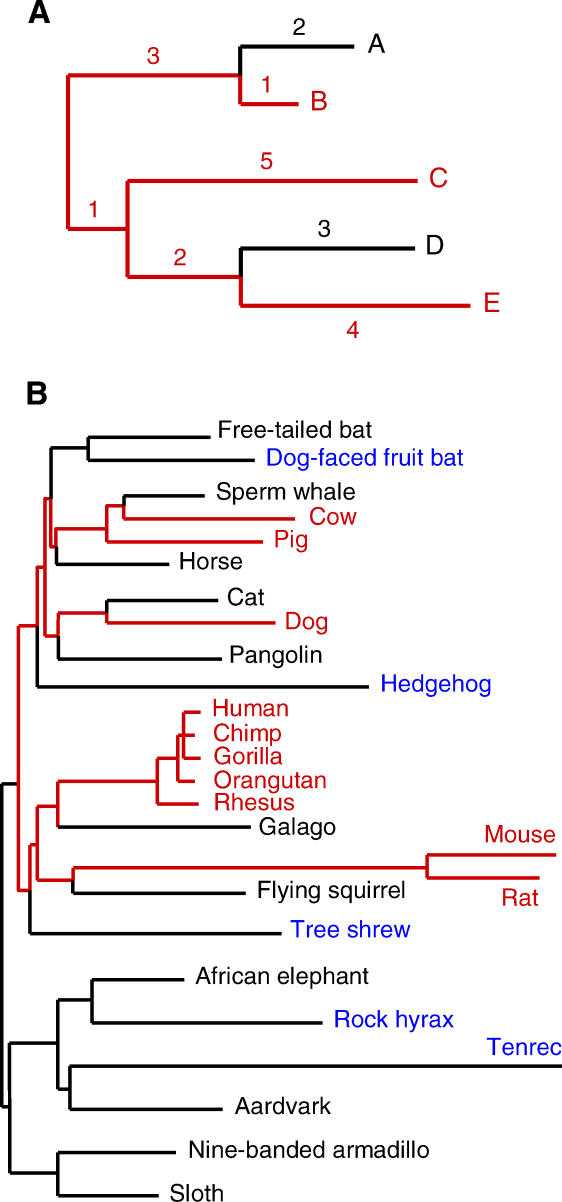
Phylogenetic Scopes and Divergence of Sets of Species (A) Phylogenetic scope comprising hypothetical species A, B, C, D, and E. Numbers are branch lengths indicating evolutionary distances (not necessarily reflecting temporal distances). The subtree connecting species B, C, and E is shown in red and has divergence 1 + 3 + 1 + 5 + 2 + 4 = 16. Applying the greedy algorithm always produces maximally divergent extensions of the original set. For example, the subsets constructed starting with B—BE (divergence 11), BCE (16), BCDE (19)—have maximum divergence among those obtainable by adding one, two, and three additional species, respectively. The series AE (12), ACE (17), ACDE (20) is optimal among all possible subsets of two, three, and four species. (B) Phylogenetic scope comprising placental mammals that have been or are being sequenced (in red) and candidates for future sequencing (derived from [17]). If five groups choose the next five targets for sequencing using the greedy strategy described in the text, the following species (in blue) will be selected (in order): (1) tenrec, (2) hedgehog, (3) rock hyrax, (4) tree shrew, (5) dog-faced fruit bat (a megabat). Within the phylogenetic scope shown, this is guaranteed to be the choice of five species that maximises the total resulting divergence. These species have recently been announced amongst targets for future sequencing [9].

In what follows, we give a simple algorithm which we prove solves this problem. We also consider, and answer, the novel question of whether different sequencing actors (groups, institutes, consortia) need to cooperate when choosing genomes: does lack of coordination and planning lead to “suboptimal” choices of genomes? While this paper assumes that optimality coincides with maximum divergence, as defined above, our results also hold for many more general species choice criteria (see Materials and Methods for details).

## Results/Discussion

Imagine adopting the following “greedy” algorithm for the divergence maximisation problem: repeatedly select one species that adds the most divergence to the previously chosen ones, until *k* species have been added. A greedy strategy might be suspected of “short-sightedness,” i.e., leading to suboptimal solutions. We can imagine realising that a better solution could have been devised if we had considered the problem of choosing all *k* species at once. Perhaps surprisingly, this cannot happen. Whatever alternative strategy we devise, no better solution than that provided by the greedy algorithm is obtained. This proposition is exemplified in Figure 1A and formally proven in Materials and Methods. Note that even when the set of species previously sequenced was not optimal, the greedy algorithm guarantees the best possible subsequent extension.

Greedy algorithms are well known in computer science and often fail to guarantee optimal solutions [14]. Our result is not only of algorithmic interest, but has consequences for real-life strategies for genome sequencing. Figure 1B shows an imaginary scenario (perhaps not too far from reality) in which the genomes of a number of placental mammals have already been sequenced, and others are candidates for future sequencing. Imagine that a number of groups each have the resources to sequence one more mammal. How should they behave in order to ensure that a maximally divergent set of species is obtained? Is some sort of cooperation necessary?

Clearly, openness regarding each group's decision is necessary, since if one decides to sequence, say, the cat, the others must avoid sequencing this or any other closely related feline. Similarly, within the framework of maximising divergence, the real-life choice to sequence the rat [2] just after the mouse [1] was far from optimal. But apart from communicating their intentions, is real cooperation among the groups necessary? Applying the result described above, it is apparent that the answer is no. If every group selfishly (“greedily”) decides to sequence the genome that at the moment of choice is the most “appealing”—i.e., adds the most divergence to the set of species already sequenced or previously chosen by the other groups—then the best possible outcome is guaranteed. Another practical consequence of the optimality of the greedy algorithm is that no planning is needed, either. Specifically, no consideration of next (or any future) year's resources is necessary when determining priorities for this year's expenditure.

The greedy algorithm also guarantees an optimal solution even when other criteria for evaluating species' importance—not only divergence—are taken into account: for example, proximity to a particularly interesting species (such as human), genome size, knowledge of the species' biology, or amenability to laboratory research [11] (see Materials and Methods for further discussion). Because of this flexibility, the optimality of the greedy strategy also applies in choosing species for purposes outside comparative genomics: clearly, for genome sequencing tout-court (even when comparison is not the first use of the genome sequence) and, interestingly, for biodiversity conservation [15,16], where divergence maximisation is also considered an important objective.

If genome (or conservation) scientists follow a seemingly short-term strategy—involving neither planning nor cooperation in the choice of future genomes for sequencing (or species for conservation)—then, provided they are open about their choices, they are guaranteed the best long-term strategy.

## Materials and Methods

### Correctness of the greedy algorithm.

A result related to ours has been independently obtained by Steel [16], whose study concentrated on its relevance in biodiversity conservation. Steel proves that the application of the greedy algorithm on a maximally divergent set of species always results in other, larger, maximally divergent sets of species. Here, we additionally prove that applying the greedy algorithm to an initial set that is not maximally divergent results in optimal extensions of the initial set.

The idea of the proof is the following. We first prove (Theorem 1; see below) that applying a greedy choice to further extend an already optimally extended set of species always results in another optimally extended set of species. Since the first step of the greedy algorithm necessarily results in an optimally extended set, subsequent steps will construct only other optimally extended sets (Corollary 1; see below). The greedy algorithm can therefore be used to construct optimal extensions of any desired size.

### Notation.

T_S_ is a tree connecting the species in set S (coinciding with its leaves). Branches in T_S_ are assumed to have non-negative lengths. Letters I, X, and Y will always denote subsets of S; *k* is a non-negative integer.

### Definitions.

The tree spanning X, denoted by T_X_, is the smallest subtree of T_S_ connecting all the species in X. A path is a sequence of adjacent branches in T_S_. The terminal path of T_X_ leading to *x* (in X), is the path from T_X−{*x*}_ to *x*. The divergence of X, denoted by δ(X), is the sum of all the branch lengths in T_X_. Y is a *k*-extension of X if Y can be obtained by adding to X *k* species not in X. X is a maximally divergent *k*-extension (*k*-MDE) of I if (a) X is a *k*-extension of I, and (b) for every *k*-extension Y of I, δ(Y) ≤ δ(X). We call a 1-MDE of X a greedy extension of X and denote it by X^+^. Note that X^+^ need not be unique, but any X^+^ will satisfy the theorem below. We will also say that X^+^ is obtained from X through a greedy step.

We now prove that the application of a greedy step to a maximally divergent extension (X) of an initial set (I) necessarily results in another maximally divergent extension (X^+^). Informally, we show that however any extension (Y) with the same size as X^+^ is constructed, a set that is at least as divergent as Y can be obtained from X by adding one species in Y to X. Therefore the greedy step, which can add any species to X—not only those in Y—will necessarily lead to a total divergence in X^+^ that is at least as great as that in Y. X^+^ therefore has maximum divergence among all its equally sized extensions of the initial set.

**Figure 2:**
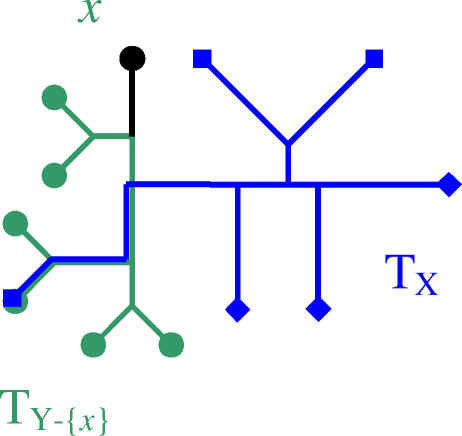
Representative Example for the Scenario Described in the Proof of Theorem 1. T_X_ is depicted in blue and T_Y-{*x*}_ in green. Species (leaves) in Y are represented by filled circles.

### Theorem 1.

Consider sets I and X, where X is a *k*-MDE of I, and 2 ≤ |X| < |S|. Then X^+^ is a (*k* + 1)-MDE of I.

Proof. Let Y be any (*k* + 1)-extension of I. By the lemma below, there exists at least one terminal path of T_Y_, leading to a leaf *x* not in X (and therefore not in I), which is completely contained in the path from T_X_ to *x* (see Figure 2). Then


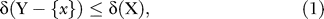


as X is a *k*-MDE of I, and

length of the path from T_Y−{*x*}_ to *x* ≤ length of the path from T_X_ to *x,*

as the second path contains the first. Thus,


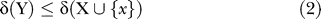


by summing the terms above. But


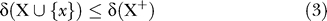


by the definition of X^+^. Therefore,


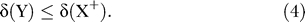


Since the last inequality holds for any (*k* + 1)-extension Y of I, X^+^ is a (*k* + 1)-MDE of I.

### Observation.

Theorem 1 claims that the greedy extension of any *k*-MDE is a (*k* + 1)-MDE, assuming that the *k*-MDE has at least two species. This assumption ensures that either I is nonempty or *k* ≠ 1. In fact, if we have both I empty and *k* = 1, the theorem is not true: in this case, any 1-extension X of the empty set has δ(X) = 0 and is maximal. However, not every X^+^ will be maximal.

### Corollary 1.

Let I be non-empty. The iterated application of any number *k* of greedy steps to I (i.e., the greedy algorithm) results in a *k*-MDE of I.

Proof. By induction: one greedy step results in the 1-MDE of I; if *h* ≥ 1 greedy steps construct an *h*-MDE of I, then by Theorem 1 one more step will construct an (*h* + 1)-MDE of I.

### Corollary 2.

Let X be a maximally divergent set of *h* species (with *h* ≥ 2). Applying the greedy algorithm to X for *k* steps results in a maximally divergent set of *h* + *k* species.

Proof. Apply Theorem 1 with I empty, and observe that *k*-MDEs of the empty set are maximally divergent sets of *k* species. It should be noted that Corollary 2 has been proven directly by Steel [16].

### Lemma.

Suppose 2 ≤ |X| < |Y|. Then there exists a leaf *x* in Y − X such that the path from T_X_ to *x* completely contains the terminal path of T_Y_ leading to *x*.

Proof. Suppose the contrary. Then, for all *x* in Y − X, either (A) T_X_ is contained in a subtree of T_S_ that departs from the terminal path of T_Y_ leading to *x*, or (B) T_X_ overlaps with the terminal path of T_Y_ leading to *x* (see Figure 3).

**Figure 3:**
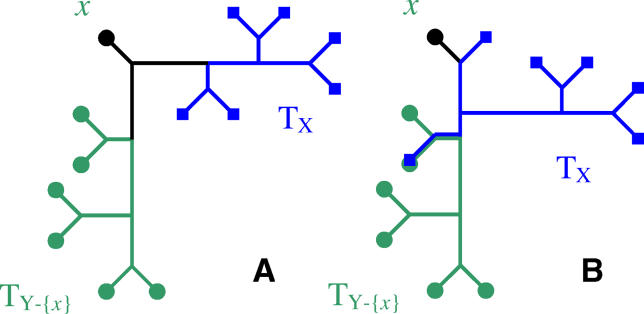
Representative Examples for Two Scenarios in the Proof of the Lemma In both examples, T_X_ is in blue and T_Y-{*x*}_ in green, and species (leaves) in Y are represented by filled circles. The two scenarios in the proof of the lemma, A and B, are illustrated correspondingly in (A) and (B), respectively.

Both (A) and (B) imply the presence of one or more leaves of X in one of the subtrees of T_S_ that depart from the terminal path of T_Y_ leading to *x*. Clearly, none of these leaves can be in Y. There is at least one of these leaves (an element of X − Y) for each terminal path of T_Y_ leading to a species *x* not in X. Since |Y| > 2, all of these terminal paths are distinct; therefore, there are exactly |Y − X| of them and at least one leaf in X − Y for each of them, i.e., we have |X − Y| ≥ |Y − X|. But this is equivalent to |X| ≥ |Y|, which contradicts the lemma's assumptions.

### Example.

For the particular case |X| = 2 and |Y| = 3, it is easy to see that the lemma holds by looking at all six possible topologically distinct cases, depicted in Figure 4.

**Figure 4:**
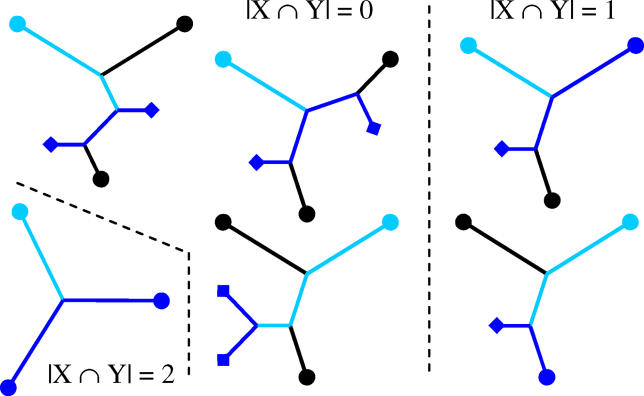
Topologically Distinct Phylogenetic Trees for Two Sets of Species, X and Y, such that |X| = 2 and |Y| = 3. X and T_X_ are depicted in dark blue, leaves in Y are denoted with circles, and a possible choice for *x* (satisfying the requirements in the lemma), with the path from T_X_ to *x,* in light blue.

### Divergence maximisation can formalise other criteria for species selection.

Evolutionary divergence is not the only criterion guiding the selection of species for sequencing [11]. The perfect example of this comes from the decision to sequence both the mouse [1] and the rat [2], which are evolutionarily relatively close. These species were chosen because they are very well known model organisms, well suited for experimental studies, and medically relevant. It is important to note that preference towards the selection of particular species—for whatever reason—can also be formalised using a divergence maximisation approach. If we extend the terminal branch leading to each species by an amount reflecting that species' estimated importance, then application of our greedy algorithm to this modified tree leads to an optimal compromise between maximising “real” evolutionary divergence and including “preferred” species.

What kind of criteria may one take into account? The mouse-rat example already suggests some of these: deep knowledge of an organism's biology should be an advantage, as should its suitability for experimental (genetic) studies. Furthermore, we might have an intrinsic interest in one particular organism in the phylogenetic scope, and therefore we will tend to select species that are closely related to it, as these will probably share many of the genetic features we are interested in. The typical example of this is the human, but in almost every phylogenetic scope a “pivotal” species can be identified, usually a traditional model organism. The pivotal species need not be extant: one could be interested in an extinct organism, for example in reconstructing ancestral sequences or genome structure [18]. Scientific reasons are not the only ones playing a role; as in every human activity, economic interests have a crucial impact, and we expect many plant and animal genomes to be selected for sequencing on the basis of potential applications in biotechnology. Finally, one should not underestimate the importance of sequencing costs, which clearly favour species with small genome sizes.

Once these criteria are somehow quantified—which is easy at least for sequencing costs or evolutionary proximity to a pivotal species—and some idea of their relative importance defined, then we can calculate for each species a “preference score” proportional to the weighted average of that species' scores under the various criteria. We can then extend each species' terminal branch by its preference score. In practice, it may not be possible to quantify these criteria or relative weights in a generally accepted manner. Nevertheless, we can imagine that some tree modified in this way could account for the evaluation of what is “appealing” in reality being influenced by more than simply evolutionary divergence. Then greedy behaviour of sequencing groups—always choosing the currently most “appealing” species—coincides with the greedy algorithm applied to this tree, and our result provides reassurance that such behaviour will lead to an optimal solution with respect to real-life evaluations.

Note that here we assumed that it is possible to formalise the sequencing “value” of a set of species in the way described above, i.e., as the divergence of a suitably constructed tree. This is not true for all conceivable criteria for evaluating species sets, but is true at least for those that can be represented as per-lineage additive measures of value. We believe that most real-life criteria for choice [11] fall into this category.
